# Left ventricular twist is load-dependent as shown in a large animal model with controlled cardiac load

**DOI:** 10.1186/1476-7120-10-26

**Published:** 2012-06-25

**Authors:** Roman A’roch, Ulf Gustafsson, Jan Poelaert, Göran Johansson, Michael Haney

**Affiliations:** 1 Department of Surgical and Perioperative Sciences, Anaesthesiology and Intensive Care Medicine, University Hospital of Umeå, 90185, Umeå, Sweden; 2 Department of Clinical Physiology, Heart Centre, University Hospital of Umeå, Umeå, Sweden; 3 Department of Anaesthesiology and Perioperative Medicine, University Hospital of Brussels, Brussels, Belgium; 4 Department of Surgical and Perioperative Sciences, Anaesthesiology and Intensive Care Medicine, University Hospital of Umeå, Umeå, Sweden; 5 Department of Surgical and Perioperative Sciences, Anaesthesiology and Intensive Care Medicine, University Hospital of Umeå, Umeå, Sweden

**Keywords:** Echocardiography, Ventricular function, Rotation, Torsion, Load

## Abstract

**Background:**

Left ventricular rotation and twist can be assessed noninvasively by speckle tracking echocardiography. We sought to characterize the effects of acute load change and change in inotropic state on rotation parameters as a measure of left ventricular (LV) contractility.

**Methods:**

Seven anesthetised juvenile pigs were studied, using direct measurement of left ventricular pressure and volume and simultaneous transthoracic echocardiography. Transient inflation of an inferior vena cava balloon (IVCB) catheter produced controlled load reduction. First and last beats in the sequence of eight were analysed with speckle tracking (STE) during the load alteration and analysed for change in rotation/twist during controlled load alteration at same contractile status. Two pharmacological inotropic interventions were also included to examine the same hypothesis in additionally conditions of increased and decreased myocardial contractility in each animal. Paired comparisons were made for different load states using the Wilcoxon’s Signed Rank test.

**Results:**

The inferior vena cava balloon occlusion (IVCBO) load change compared for first to last beat resulted in LV twist increase (11.67° ±2.65° vs. 16.17° ±3.56° respectively, p < 0.004) during the load alteration and under adrenaline stimulation LV twist increase 12.56° ±5.1° vs. 16.57° ±4.6° (p < 0.013), and though increased, didn’t reach significance in negative inotropic condition. Untwisting rate increased significantly at baseline from −41.7°/s ±41.6°/s vs.−122.6°/s ±55.8°/s (P < 0.039) and under adrenaline stimulation untwisting rate increased (−55.3°/s ±3.8°/s vs.−111.4°/s ±24.0°/s (p < 0.05), but did not systematically changed in negative inotropic condition.

**Conclusions:**

Peak systolic LV twist and peak early diastolic untwisting rate are load dependent. Differences in LV load should be included in the interpretation when serial measures of twist are compared.

## Background

Normal heart function incorporates an aspect of twisting during systole and ejection [1,2], and untwisting during relaxation and diastole [3,4]. When ventricular function is disturbed, the normal twist or wringing action can be affected. Reports have suggested importance of left ventricular rotation as an indicator of cardiac performance [5,6]. Some areas where twist has been assessed in the evaluation of heart disease include myocardial infarction [7], heart failure [8], regional dyssynchrony [9], and valve disease [10]. Normal amounts of LV twist in healthy individuals have been recently reported [11,12], and some effects of aging on LV twist have been reported [13-15]. Routine application of assessment of ventricular twist to clinical patient problem solving is not yet widely established, though much work has been recently published to try to validate different ventricular circumferential motion derived parameters in many patient populations.

The relation between ventricular twist and heart function is still not entirely understood. There are suggestions that the amount of ventricular twist during systole is related to not just systolic function and well-being, but also to loading conditions [16] and LV volume [17]. Other reports, on the other hand, suggest that LV twist is unaffected by changes in load (preload or afterload) [18,19].

While there have been suggestions of a strong relation between twist/untwist and load [20,21], no report to date has definitively determined if twist is load-dependent or not. We hypothesised that LV twist would change if the left ventricle was exposed to controlled changes in loading while at the same inotropic status. We further hypothesised that twist and untwist-rate would change when LV function (contractility) was altered experimentally. We aimed to characterize the effects of acute load change and change in inotropic state on rotation parameters as a measure of LV contractility.

## Methods

With approval of the Ethics Committee of Umeå University, and in conformation with the Guide for the Care and Use of Laboratory Animals (National Academy of Sciences, 1996, USA) seven juvenile Yorkshire/Hampshire pigs (mean weight 36.6 ± 3.7 kg; SD) were anaesthetised and instrumented using methods that have been well described previously [22]. In brief, the animals were premedicated with ketamine 10 mg·kg^−1^, xylazine 2.2 mg.kg^−1^, and atropine 50 μg.kg^−1^ i.m. Anaesthesia was induced with pentobarbital 12 mg.kg^−1^ i.v. and maintained by a continuous infusion of pentobarbital 5 mg.kg^−1^.h^−1^, midazolam 0.3 mg.kg^−1^.h^−1^ and fentanyl 20 μg.kg^−1^.h^−1^. After tracheotomy, animals were ventilated (Evita4, Dräger, Germany) to achieve normoxia and normocapnea (Marquette Solar 8000, GE Healthcare, Stockholm, Sweden). Intravenous fluids were administered: Ringer’s Acetate 15 ml·kg^−1^ h^−1^ throughout the study period. Arterial and venous catheters were placed through cutdowns to the jugular and carotid vessel systems. Arterial line and central venous catheters, including a 7 F Swan-Ganz catheter (Optimetrix, Abbott, Illinois, USA) was placed first. A combined pressure-conductance catheter, with 12 electrodes and 8 mm spacing in between electrodes (CA-71083-PN, CD Leycom, Zoetermeer, Holland), was placed in the long axis of the LV with the help of fluoroscopy. A 7.5 F balloon occlusion catheter (Vascular Technologies, Solna, Sweden) was placed in the inferior vena cava in order to facilitate a controlled transient restriction of venous return during measurement periods. Although it has to be mentioned that very large and abrupt loading changes associated with transient inferior vena cava balloon occlusion (IVCBO) can result in short-term alterations in sympathetic tone and ventricular interaction, which can influence the pressure-volume (PV) relationship, a careful load alteration within physiological or normal load ranges, this method belongs to an established method comparing LV function under changing load [23].

The conductance catheter allows continuous online measurements of LV pressure and volume and the method of left ventricular volume measurement with dual field conductance volume measurements is well described elsewhere [24]. The conductance catheter was connected to a signal conditioning-amplifier set to dual-field mode (Leycom Sigma 5DF, CD Leycom, Zoetermeer, The Netherlands). Parallel conductance and flow reference ratio were determined for LV volume calibration [25]. Left ventricular pressure (Sentron, Roden, The Netherlands) and conductance data were recorded with a frequency of 250 Hz (PC Conduct, Cardiodynamics, Zoetermeer, The Nether-lands). All circulatory parameters were recorded digitally and analyzed (Acknowledge, Biopac Systems, Santa Barbara, California). Pressure-volume data analysis was performed with custom-made software. Cardiac performance was assessed by heart rate, stroke volume, end-diastolic volume, end-systolic volume, cardiac output, and stroke work. Systolic load-dependent LV function was determined by the EF, end-systolic pressure, maximal rate of LV pressure increase (dP/dt_max_), and load-independent LV function by the linear slope of the end-systolic PV relationship, defined as end-systolic elastance (EES) and PRSW. Diastolic load-dependent LV function was assessed by the LV end-diastolic pressure (LVEDP), isovolumic relaxation time constant (tau), maximal rate of LV pressure decrease (dP/dt_min_) and ratio of end-systolic elastance/arterial elastance (EES/EA) [26]. Echocardiographic recordings of short-axis images at two levels, basal and apical, were done with a frame rate of 65–80 per second using an ultrasound system (Vivid 7, GE Healthcare, Horten, Norway). All recordings have been done by transthoracic approach in order to maintain myocardial/pericardial physiology. The basal level was obtained at the tips of mitral valve leaflets. The apical level was defined just proximal to the level with LV luminal obliteration at the end-systolic period and no visual papillary muscle. Baseline registrations were collected before starting the data collection regarding to the protocol. Additionally, apical four chamber views and blood flow at inflow and outflow areas of LV were also recorded using pulsed wave Doppler. The time intervals were measured from the Q wave start to aortic valve opening and closure (AVO and AVC, respectively) from trans-aortic Doppler recordings, and time to mitral valve opening (MVO) from trans-mitral Doppler, with timing help from the ECG. Rotation at 2 levels was analysed offline using a speckle tracking software (2D-strain, EchoPac 8, GE Healthcare, Horten, Norway).

### Protocol

Each measurement sequence was recorded during a period of apnea with 0 cm H2O airway pressure. The inferior vena cava balloon was inflated, and progressive beat-to-beat decreases in left ventricular volume and pressure were recorded. Beats selected for analysis from the balloon inflation period were those where there was a progressive beat by beat decrease in both LV end-diastolic and end-systolic volume and pressure at the beginning and end of the sequence. Each measurement was collected at rest, and then during adrenaline infusion with target pulse rate raise of at least 20% from the baseline. After a second rest period, 30 min after discontinuation of adrenaline infusion, a slow injection of metoprolol 40 mg and verapamil 15 mg was administrated, together with infusion of phenylephrine to counterbalance the vascular effects of verapamil and keep the blood pressure stable. Measurements were then collected during cardiovascular steady-state conditions.

### Analysis, ventricular twist

Speckle tracking analysis was used to measure LV rotation and LV twist as previously described [27]. The analysis was performed off-line by a single observer (RA) with no reference to haemodynamic data at the time of analysis. Analysis was performed using EchoPac software (GE Healthcare, Horten, Norway). Endocardium was traced, and the region of interest (ROI) was adjusted to fit most of the left ventricle in the short axis view without including the pericardium. The average LV rotation and rotational velocity profile at base and apical levels were measured (GE Echopac 8, Horten, Norway), and then LV twist was calculated as the net difference between LV rotational angles obtained from maximal basal (clockwise rotation) and maximal apical (counter-clockwise rotation) short-axis planes. Peak early diastolic untwist-rate was defined as the peak untwisting velocity during IVRT (Isovolumic relaxation time = period from AVC to MVO).

### Statistics

All data are presented as mean ± 95% confidence intervals. Paired measurements were tested for differences using Wilcoxon’s Signed Rank test in the case of first and last beats in a preload alteration sequence, and for increase or decrease from preceding measure for systolic function parameters derived from preload alteration sequences (comparison of 2 beats). A p value < 0.05 was considered statistically significant.

## Results

### General hemodynamics

**Figure 1:**
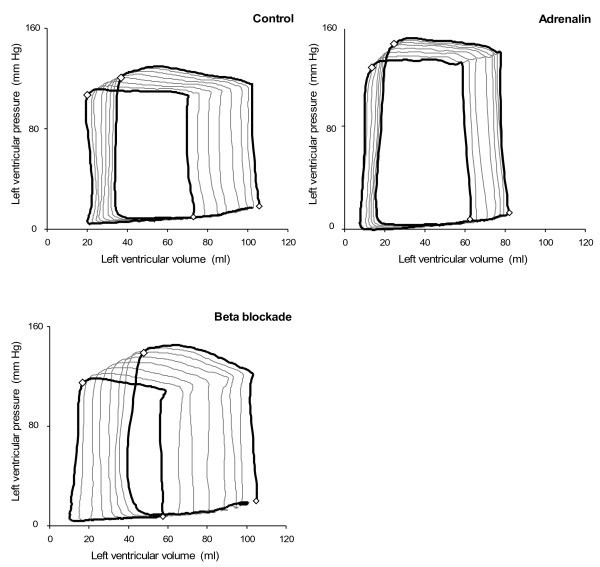
Representative vena cava occlusion sequence is demonstrated in pressure-volume diagrams with the first and last beats in the sequence shown to demonstrate the loading conditions, first at baseline, then as well as during experimentally (pharmacologically) manipulated inotropic conditions, positive inotropic condition (adrenaline) and negative inotropic condition (beta blockade).

**Figure 2:**
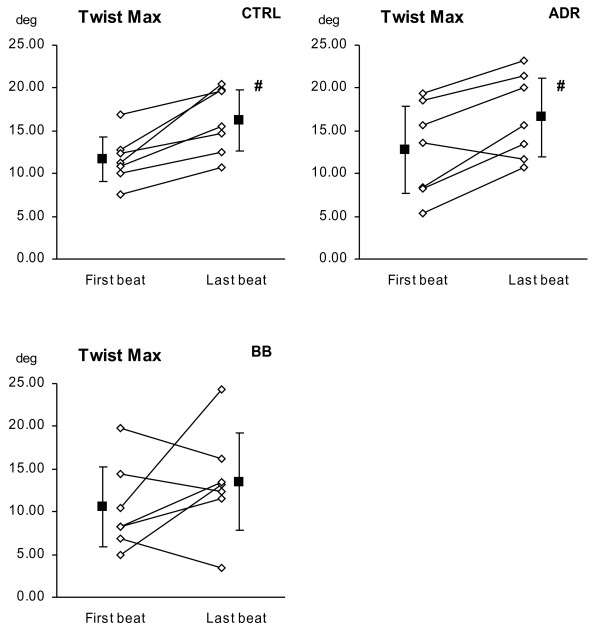
** LV twist changes for the first and last beat in a sequence of eight consecutive beats in a vena cava occlusion manoeuvre were analysed by speckle tracking imaging.** Left ventricular twist increased significantly in control group and in positive inotropic condition. There was a trend to increase but no significance was reached in negative inotropic condition. Both individual and grouped values are presented. CTRL = control group, ADR = adrenaline group, BB = beta-blockade group. # = p < 0.05 using Wilcoxons Signed Rank test vs. first beat.

**Figure 3:**
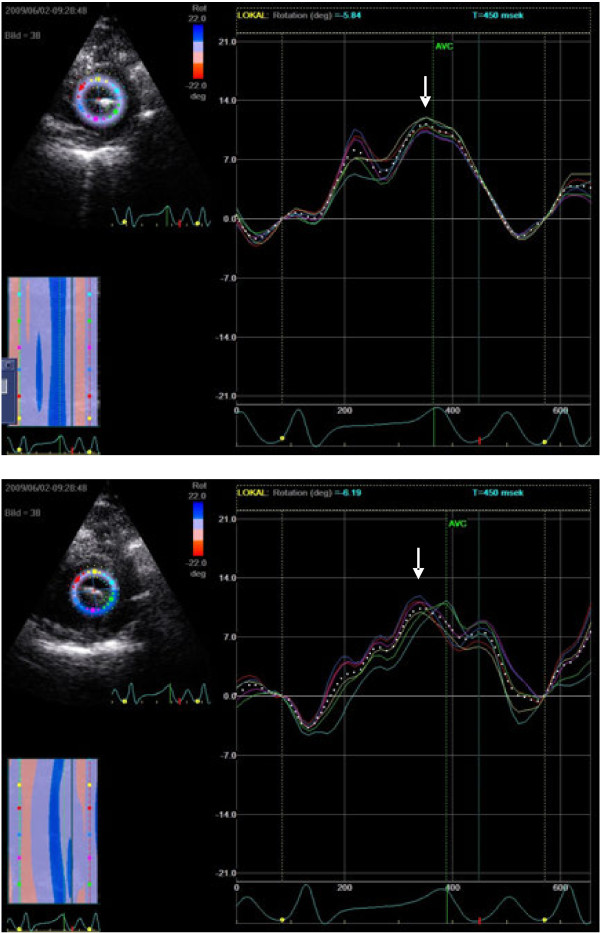
** Representative images of counterclockwise apical rotation for the first and last beat in a vena cava occlusion sequence by speckle tracking imaging.** Rotation in individual segments and mean values are presented. Maximal apical rotation (arrow) increased from 5.8 to 6.2°.

Results were collected from 7 animals that completed the protocol, with the controlled IVCBO load reduction, and for all 3 inotropic conditions, with simultaneous echocardiographic/speckle measurements together with LV pressure volume results for each sequence. General circulatory conditions (Table 1) show that end-diastolic volumes and pressures as well as end-systolic pressures as indicators of circulatory well-being were unchanged throughout the experimental protocol. Load changes were achieved by the IVCBO where the first and last beats in the sequences (Figure 1, 2 and 3) are analysed (Table 1) and a clear load alteration (reduction) is shown (end-diastolic pressures and volumes, as well as end-systolic pressure and volume), and this was performed with all 3 inotropic conditions. The 3 inotropic conditions (Table 2) were analysed for parameters derived from the controlled preload alteration sequences (end-systolic elastance, preload recruitable stroke work, maximal instantaneous power/end-diastolic volume, and dp/dt_max_/end-diastolic volume) showed differences from control for the inotropic experimental interventions, with increased and decreased contractile status demonstrated for the inotropic interventions. Concerning single beat parameters and load, the systolic parameters, including stroke work and ejection fraction, showed decreases related to decreased load (Table 1). Diastolic function parameters, including tau and pressure half-time (PHT), did not show large changes related to the load alteration with IVCBO in the control resting stage, though there were tendencies for tau to in-crease and PHT to decrease during load reduction in the inotropic treatment groups (Table 1), while dP/dt_min_ decreased with decreasing load (Table 1) as well as changing in relation to inotropic intervention.

**Table 1:** Left ventricular parameters during vena cava occlusion, first and last beat

**CONTROL**		First beat	Last beat	
Ves	(mL)	42.0 ± 7.9	21.3 ± 6.4	#
Ved	(mL)	100.3 ± 12.7	69.2 ± 11.0	#
Pes	(mm Hg)	118.6 ± 4.5	100.7 ± 6.6	#
Ped	(mm Hg)	12.0 ± 4.3	1.9 ± 3.7	#
dPdtMax	(mm Hg/s)	2797 ± 429	3035 ± 526	#
EF	(%)	61.9 ± 6.2	70.5 ± 7.0	#
SW	(mm Hg mL)	7097 ± 1385	4544 ± 891	#
Tau	(ms)	34.30 ± 2.77	34.10 ± 3.61	
PHT	(ms)	20.85 ± 2.12	19.06 ± 2.56	
dPdtMin	(mm Hg/s)	−2776 ± 186	−2347 ± 158	
**ADRENALINE**				
Ves	(mL)	32.0 ± 13.10	11.0 ± 7.4	#
Ved	(mL)	96.0 ± 32.4	62.9 ± 20.3	#
Pes	(mm Hg)	123.1 ± 18.0	98.0 ± 12.3	#
Ped	(mm Hg)	14.0 ± 6.9	3.0 ± 4.6	#
dPdtMax	(mm Hg/s)	5389 ± 1519	5416 ± 1538	
EF	(%)	72.6 ± 7.2	85.0 ± 9.7	#
SW	(mm Hg mL)	8725 ± 2833	5822 ± 2415	#
Tau	(ms)	24.12 ± 2.87	31.11 ± 2.80	#
PHT	(ms)	14.92 ± 2.24	16.54 ± 1.66	
dPdtMin	(mm Hg/s)	−3454 ± 775	−2314 ± 392	
**BETA-BLOCKADE**				
Ves	(mL)	52.2 ± 9.7	23.5 ± 10.9	#
Ved	(mL)	105.1 ± 20.0	62.0 ± 14.3	#
Pes	(mm Hg)	115.5 ± 12.7	91.7 ± 9.3	#
Ped	(mm Hg)	12.9 ± 5.6	−0.6 ± 4.3	#
dPdtMax	(mm Hg/s)	1925 ± 527	1909 ± 473	
EF	(%)	55.4 ± 6.5	69.5 ± 16.7	#
SW	(mm Hg mL)	5907 ± 1675	3375 ± 826	#
Tau	(ms)	47.14 ± 20.61	36.84 ± 7.06	#
PHT	(ms)	26.40 ± 6.62	20.76 ± 5.06	#
dPdtMin	(mm Hg/s)	−2244 ± 414	−1992 ± 341	

Ves = end-systolic volume; Ved = end-diastolic volume; Pes = end-systolic pressure; Ped = end-diastolic pressure; dPdt max = maximal rate of pressure increase; EF = ejection fraction; SW = stroke work; Tau = isovolumic relaxation time constant; PHT = pressure half time during diastole: dPdt min = maximal rate of pressure decrease. Data are presented as mean ± 95% confidence intervals, n = 7. # = p < 0.05 using Wilcoxons Signed Rank test vs. first beat.

**Table 2:** Left ventricular parameters during control, adrenaline and beta-blockade

		Control	Adrenaline		Beta blockade	
*Apnea measurement*						
HR	(bpm)	125 ± 19	147 ± 29		101 ± 12	
SV	(mL)	60 ± 9	65 ± 26		50 ± 17	#
CO	(L/min)	7.4 ± 1.3	9.3 ± 3.8		5.0 ± 1.7	#
Ves	(mL)	40.9 ± 4.4	37.8 ± 15.3		55.8 ± 10.4	#
Ved	(mL)	96.5 ± 8.4	100.3 ± 31.9		101.6 ± 22.6	
Pes	(mm Hg)	119.0 ± 7.4	121.8 ± 5.1		106.7 ± 16.0	
Ped	(mm Hg)	15.6 ± 5.2	15.8 ± 6.7		19.6 ± 5.7	
dPdt max	(mm Hg/s)	2830 ± 430	5483 ± 14	#	1726 ± 594	#
EF	(%)	62.0 ± 6.1	64.9 ± 14.0		47.7 ± 8.0	#
SW	(mm Hg mL)	6744 ± 1054	8254 ± 3061		4608 ± 1858	#
dPdt min	(mm Hg/s)	−2889 ± 193	−3483 ± 680		−2051 ± 553	
Tau	(ms)	33.9 ± 4.8	24.5 ± 2.2	#	48.6 ± 25.9	
PHT	(ms)	20.8 ± 2.4	15.6 ± 2.1	#	32.0 ± 14.6	#
PWRmax	(mm Hg mL/s)	50210 ± 9173	72286 ± 30668		35756 ± 10130	#
PWRmax/EDV^2^		5.46 ± 1.15	8.30 ± 4.24		3.62 ± 0.94	#
dPdt/EDV	(mm Hg/s/mL)	29.7 ± 5.8	63.1 ± 29.5	#	18.0 ± 9.8	#
*VCBO measurement*						
Ees	(mm Hg/mL)	0.95 ± 0.32	1.65 ± 1.00	#	1.03 ± 0.61	
PRSW	(mm Hg)	83.4 ± 12.5	100.1 ± 28.5		56.6 ± 18.3	#

HR = heart rate; SV = stroke volume; CO = cardiac output; Ves = end-systolic volume; Ved = end-diastolic volume; Pes = end-systolic pressure; Ped = end-diastolic pressure; dPdt max = maximal rate of pressure increase; EF = ejection fraction; SW = stroke work; Tau = isovolumic relaxation time constant; PHT = pressure half time during diastole: dPdt min = maximal rate of pressure decrease; PWRmax = maximal power; Ees = end-systolic elastance; PRSW = preload recruitable stroke work. Data are presented as mean ± 95% confidence intervals, n = 7. # = p < 0.05 using Wilcoxons Signed Rank test vs. Control.

### T*wist and load*

The main results here were that the load decrease, from first to last beat during VCBO, was associated with LV twist increase (11.67° ±2.65° vs. 16.17° ±3.56°, p < 0.05), as shown for the control group with no inotropic intervention (Figure 1, Table 3). While neither apical nor base rotation by themselves changed significantly in relation to load change, there were tendencies in both which, when combined to express twist, demonstrated increases both for maximal twist as well as twist at aortic valve closure. This increase in twist during load reduction was also significant during the positive inotropic condition, though the same tendency did not reach significant levels in the negative inotropic condition (Table 3).

**Table 3:** Echocardiographic parameters during vena cava occlusion

**CONTROL**		First beat	Last beat	
Twist_AVC	(deg)	9.71 ± 2.53	13.07 ± 3.72	#
Twist_MAX	(deg)	11.67 ± 2.65	16.17 ± 3.56	#
Rot_AVC apex	(deg)	7.00 ± 2.01	8.86 ± 3.92	
Rot_AVC base	(deg)	−1.57 ± 2.65	−3.61 ± 3.02	#
Rot_Max apex	(deg)	8.10 ± 2.29	10.70 ± 3.17	
Rot_AVC base	(deg)	−2.40 ± 3.28	−5.47 ± 2.58	#
IVR_Diff	(deg)	0.61 ± 1.44	4.71 ± 3.10	#
Rot_rate	(deg/s)	−41.7 ± 41.6	−122.6 ± 55.8	#
**ADRENALINE**				
Twist_AVC	(deg)	8.23 ± 4.06	12.13 ± 3.55	#
Twist_MAX	(deg)	12.56 ± 5.10	16.57 ± 4.60	#
Rot_AVC apex	(deg)	4.00 ± 3.35	7.33 ± 3.32	#
Rot_AVC base	(deg)	−4.23 ± 2.86	−1.80 ± 5.03	
Rot_Max apex	(deg)	7.51 ± 3.75	10.66 ± 3.39	
Rot_AVC base	(deg)	−5.24 ± 3.37	−5.91 ± 2.51	
IVR_Diff	(deg)	1.08 ± 0.93	2.54 ± 1.73	
Rot_rate	(deg/s)	−55.3 ± 13.8	−111.4 ± 24.0	#
**BETA-BLOCKADE**				
Twist_AVC	(deg)	9.33 ± 4.52	11.67 ± 5.46	
Twist_MAX	(deg)	10.60 ± 4.66	13.51 ± 5.74	
Rot_AVC apex	(deg)	7.19 ± 4.61	6.04 ± 5.35	
Rot_AVC base	(deg)	−1.46 ± 1.60	−4.01 ± 3.06	
Rot_Max apex	(deg)	7.81 ± 4.08	7.14 ± 5.69	
Rot_AVC base	(deg)	−2.53 ± 2.15	−5.14 ± 3.58	
IVR_Diff	(deg)	1.07 ± 1.42	2.53 ± 3.85	
Rot_rate	(deg/s)	−53.1 ± 40.5	−48.3 ± 46.5	

Twist AVC- net difference of LV rotation between apical and basal short axis planes at aortic valve closure; Twist Max- maximal LV rotation difference; Rot AVC apex- rotation at apical short axis plane at aortic valve closure; Rot AVC base- rotation at basal short axis plane at aortic valve closure; Rot Max apex- maximal rotation at apical short axis plane; Rot Max base-maximal rotation at basal short axis plane; IVR Diff- difference in untwisting under IVR period; Rot rate- untwisting rate under IVR period. Data are presented as mean ± 95% confidence intervals, n = 7. # = p < 0.05 using Wilcoxon’s Signed Rank test vs. first beat.

### Untwist and load

The main parameter for peak untwisting (rotation rate during early diastole) increased significantly during load reduction (first beat, resting load −41.7°/s ± 41.6°/s vs. last IVCBO beat −122.6°/s ± 55.8°/s, p < 0.05) expressed as more rapid untwisting in these lower load conditions where systolic twist amount was greater. There were small but significant decreases in dP/dtmin with load reduction where end-systolic pressure was also lower in these last (load-reduced) beats compared to the resting load first beat.

### Positive and negative inotropic conditions

While there were clear changes in measured parameters reflecting contractile status related to the positive and negative inotropic interventions (Table 2), the inotropic interventions did not change the pattern of response for twist and untwist as measured at first and last beats during the unload sequence (Table 3). This was demonstrated as a trend in the negative inotropic intervention, not reaching statistical significance.

## Discussion

The main findings were that twist and untwist were found to be load-dependent in this experimental model where both load and contractile conditions were well controlled. Also, this load-dependence for twist and untwist seemed to be present in the setting of altered inotropy, though the findings were less clear during the negative inotropic intervention. These results confirm those published by Gibbon et al. [17], though now in our study addressing a more modern assessment of twist. There have been previous suggestions that twist is sensitive to both load and contractility [28,29]. In one early study, apical rotation was noted to be largely a function of volume in an open chest animal model [17]. Some clinical [18,19] and experimental studies [30] have supported the idea that left ventricular torsion is relatively insensitive to load and volume, though responsive to inotropic interventions. Some recent findings in clinical material suggest that torsion or twist is load-sensitive, though this was tested using methodology where load alterations were not completely controlled [21,31]. Our study confirms that twist is exquisitely load sensitive even in physiological load ranges, and that the effect of load alteration seems to be stronger than the effect of inotropic intervention. Our results also included observations of increased rate of untwist in beats with lower load, agreeing with some previous findings [17,31] though not all studies of this [21].

### Methodological aspects

Recent studies of left ventricular torsion have employed echocardiographic speckle tracking based on validation versus magnetic resonance imaging of torsion [27,32], though the earlier reports have employed other methods. Echocardiographic and speckle images were reliably obtained using this transthoracic approach, where there was analysis possible for all views in all 7 animals. There was no technical difficulty in obtaining adequate images. The LV apical short-axis images were subject to through-plane motion, and this can have affected the accuracy of the measurement of LV rotational parameters [33].

The model included an undisturbed thorax together with separate controlled experimental interventions for load and contractility, allowing examination of each load and contractile interventions individually as well as allowing assessment of the interaction of load and contractility. Even though there were both positive and negative inotropic interventions in this large animal model, these results reflect normal (non-diseased) heart function.

The strength of this model and these findings is that the model separates load conditions from ventricular performance. In this way, 2 beats, in the same sequence with different loads but with the same contractile status, can be compared for a variety of ventricular performance indicators, including twist and untwist. Furthermore, this model allowed assessment of load effects from beat to beat also in the setting of experimentally altered contractile status. The distinctive value of this study lies in simultaneously using controlled load-specific interventions and detailed measurement of absolute load and ventricular performance in shoving load dependency of LV twist and untwist rate.

While it has been shown clearly that contractility affects twist, [28] it has not been clear demonstrated whether these changes are mediated through changes in volumes or rather primarily contractile effect. Our results suggest that both load and contractile status can affect twist and untwist in the healthy heart setting, but the effects of load seem to be more prominent. The implications of these findings are that serial measurements of torsion in individuals need to be indexed particularly for load, but also for contractile status.

In this model, ventricular volumes, pressures, and relative inotropic status were very carefully controlled, though these parameters are not generally available during routine bedside cardiovascular examination. Still, in order for twist or torsion to be used in the serial assessment of ventricular function, there needs to be a systematic coupling of twist findings to some indices of load and contractile status. While twist and untwist are not part of the standard basic echocardiographic assessment by today’s community standard, twist or torsion and untwist are increasingly being used as a complementary assessment of ventricular function [34,35]. Some means of reliably indexing for load needs to be established in order for twist or untwist to become highly reliable in judging change in heart function for individuals in a clinical setting.

In summary, these results showed that LV-twist and untwisting rate are strongly dependent on load. We conclude that before left ventricular twist and untwist can be used as routine clinical tool for serial quantitative assessment of systolic and diastolic LV dysfunction, they need to be interpreted in the context of load.

## Competing interests

All authors declare that they have no competing interests concerning this study.

## Authors’contributions

All authors participated in the initiation and design of the study. All authors except JP participated in the experiments and data collection.All authors participated in the analysis of the results. All authors read and approved the final manuscript.
